# First record of hydrozoan genera *Eucheilota* McCrady, 1859 and *Mitrocomella* Haeckel, 1879 and species *Porpitaporpita* (Linnaeus, 1758) and *Physaliaphysalis* (Linnaeus, 1758) in Bali, Indonesia

**DOI:** 10.3897/BDJ.9.e76001

**Published:** 2021-12-03

**Authors:** Widiastuti Widiastuti

**Affiliations:** 1 Department of Marine Science, Faculty of Marine Science and Fisheries, Udayana University, Bali, Indonesia Department of Marine Science, Faculty of Marine Science and Fisheries, Udayana University Bali Indonesia

**Keywords:** *Eucheilota*, *Mitrocomella*, *Porpitaporpita*, *Physaliaphysalis*, Hydromedusae, Bali

## Abstract

This report documents the first record of the genera *Eucheilota* and *Mitrocomella* and species *Porpitaporpita* and *Physaliaphysalis* in Bali, Indonesia, based on observed occurrences in different times and sites. The coincidence of the annual stranding of *Physaliaphysalis* in the east Bali and south Java coasts during the monsoon periods in Indonesia suggests a link with the upwelling events in the areas. However, more work is needed to analyse this phenomenon and study the occurrences of other Hydromedusae due to the limited data on hydrozoans in Indonesian waters. Overall, this report provides primary data to contribute to the general understanding of the biodiversity of marine organisms in Indonesia.

## Introduction

Class Hydrozoa (Phylum Cnidaria) encompasses two subclasses, Hydroidolina and Trachylinae. Hydroidolina contains the orders Anthoathecata, Leptothecata and Siphonophorae, with 118 families, whereas Trachylinae contains the orders Actinulida, Limnomedusae, Narcomedusae and Trachymedusae with a total of 28 families. Many hydrozoans have not been properly identified as species (Miglietta et al. 2008). Precise identification often requires observations of a live adult specimen and knowledge of the complete life stage. However, the small size of the medusa makes it easy to escape from plankton net/collecting tools and the similar appearance of the juvenile medusa between species and genera renders identification challenging. Despite the vast diversity of this group, hydrozoan medusae (Hydromedusae) play an important role in shaping marine ecology through their feeding habit. They serve as a potential predator of fish eggs and larvae and as a competitor for other marine organisms that compete for similar prey with them, such as zooplanktivores (Purcell 2003, Marques et al. 2017). The ability to survive in various environmental conditions such as low ecological quality, raises their capacity to predate and compete. Therefore, it is necessary to identify the species and their roles in the marine ecosystem.

Most hydrozoan species reported are native to tropical and temperate seas in the Pacific, Atlantic and Indian Oceans. However, only species from the Mediterranean Sea are well studied (Boero et al. 2005, Zenetos et al. 2005, Gravili et al. 2013). According to the map of the world's study areas of jellyfish by Brotz et al. (2012), there is limited information on jellyfish in the seas around Southeast Asia, despite the high biodiversity in the region. Studies have reported that the diversity of jellyfish in Indonesian waters mainly consists of scyphozoans: *Crambionemastigophora* in Saleh Bay, Nusa Tenggara Island (Asrial et al. 2015), *Crambionellahelmbiru* in southern waters of Central Java (Nishikawa et al. 2015), *Mastigiaspapua*, *Cassiopeiaornata* and *Aureliaaurita* in marine Lakes in Berau region, East Kalimantan, as well as Cubozoa (*Tripedaliacystophora*) (Cleary et al. 2016). Regarding hydrozoan diversity in Indonesia, order Filifera has been reported in Bali waters by Cairns and Hoeksema (1998) and the polyp of *Clytialinearis* in Kei Islands has been described by Schuchert (2003), but they are mainly in polyp forms.

The limited data on jellyfish diversity around Indonesian waters, particularly for Hydromedusae, is not only due to the inconspicuous presence of this organism, but also likely related to the developing Indonesian economy compared to other countries in the region, thus leading to neglect in understanding the local marine environment. This study reports the observations of four Hydromedusae, *Eucheilota* McCrady, 1859 from the Lovenellidae family, *Mitrocomella* Haeckel, 1879 from the Mitrocomidae family, *Porpitaporpita* (Linnaeus, 1758) from the Porpitidae family and *Physaliaphysalis* (Linnaeus, 1758) from the Physaliidae family, in Bali waters. This report provides primary data to contribute to the species checklist of marine organisms in Indonesia.

## Results

**Figure 1.**
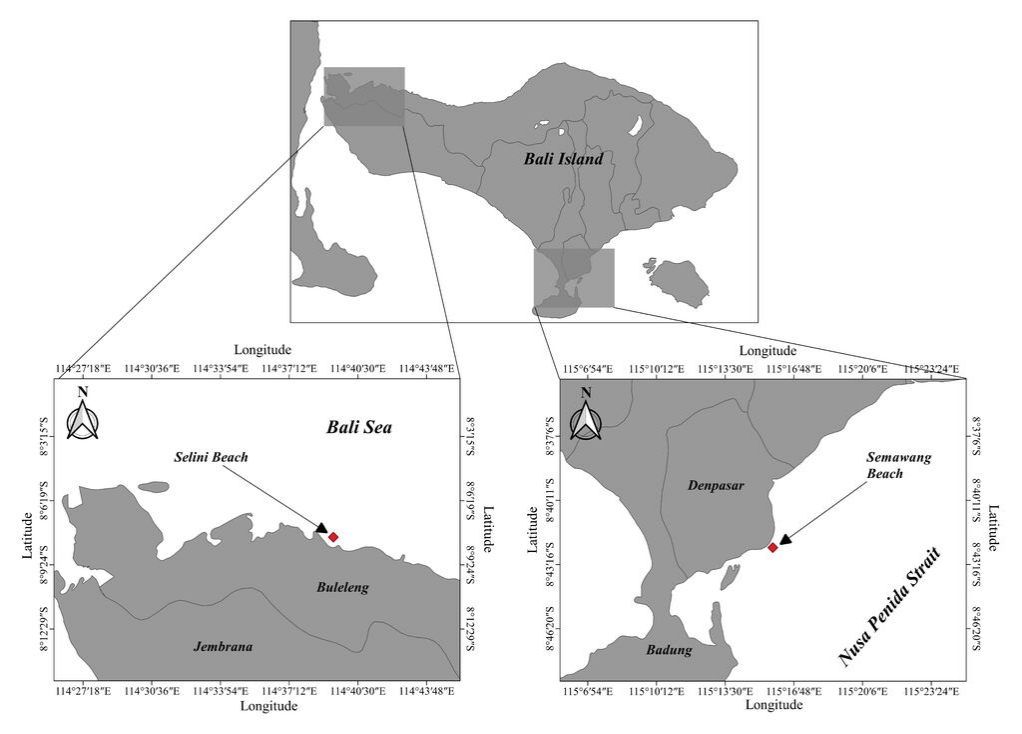
Map showing the *Porpitaporpita* and *Physaliaphysalis* (115°15'49.02156"E, 8°42'29.11284"S), *Eucheilota* and *Mitrocomellabrownei* (114°39'25.67196"E, 8°8'25.40004"S).

Four Hydromedusae were observed at different times and sites (Fig. 1). The observations of *Porpitaporpita* and *Physaliaphysalis* were based on a beach stranding in Semawang Beach (115°15'49.02156"E, 8°42'29.11284"S) on 8 July 2020, whereas *Eucheilota* and *Mitrocomella* were collected using a beaker from a boat that was moving relatively slowly along Selini Beach on 3 April 2021 (114°39'25.67196"E, 8°8'25.40004"S). The specimens were collected, photographed and identified to the genera level for *Eucheilota* and *Mitrocomella* and species-level for *Physaliaphysalis* and *Porpitaporpita*.

**Figure 2.**
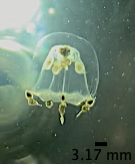
*Eucheilota* spotted on Selini Beach, Buleleng Regency, Bali Island. Lateral view of the medusae. Note the four perradial bulbs with a distinct black spot.

**Figure 3.**
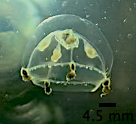
*Eucheilota* spotted on Selini Beach, Buleleng Regency, Bali Island. Lateral view of the medusae.

**Figure 4.**
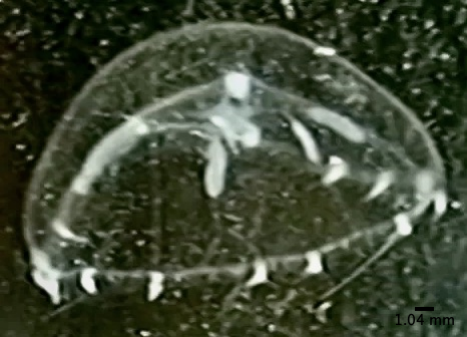
*Mitrocomella* spotted on Selini Beach, Buleleng Regency, Bali Island. Lateral view of the medusae.

**Figure 5.**
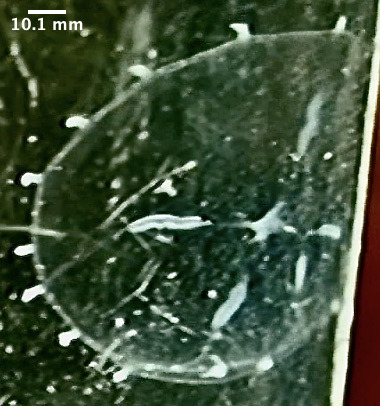
*Mitrocomella* spotted on Selini Beach, Buleleng Regency, Bali Island. Ventral view of the medusae.

**Figure 6.**
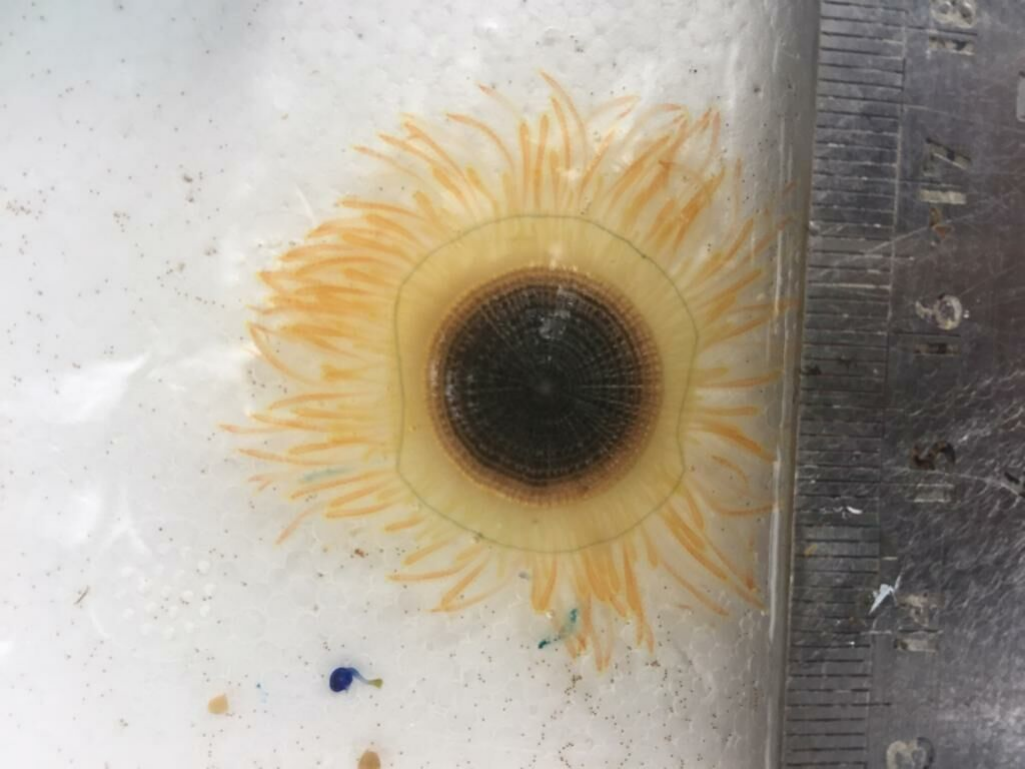
Yellow individual of *Porpitaporpita*.

**Figure 7.**
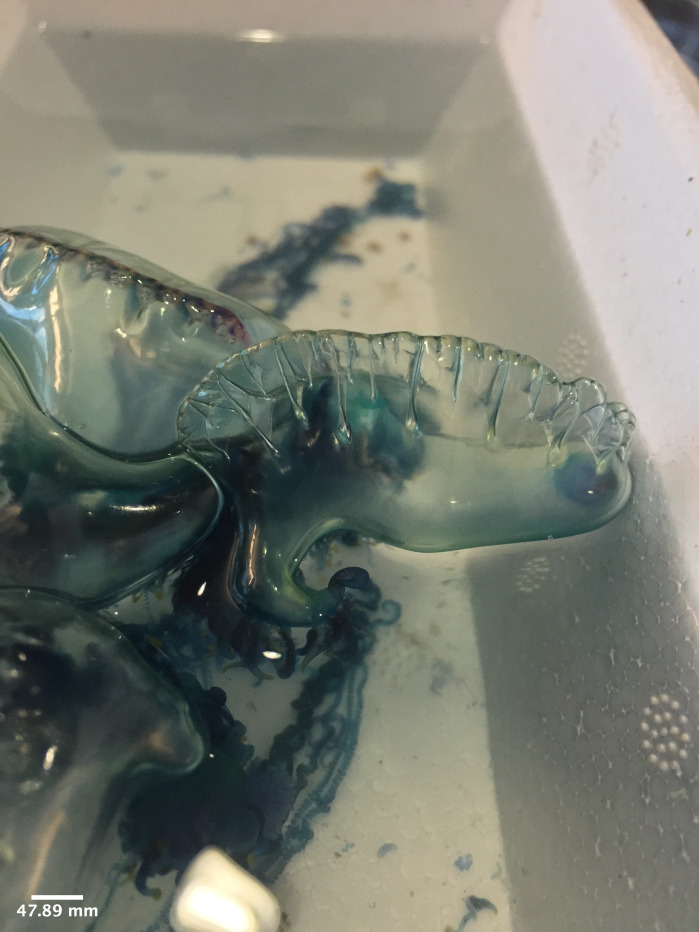
*Physaliaphysalis* washed ashore in Semawang Beach, Denpasar City, Bali Island. Note the lateral view with the pneumatophore (gas-filled float) and zooids attached to the ventral side.

**Figure 8.**
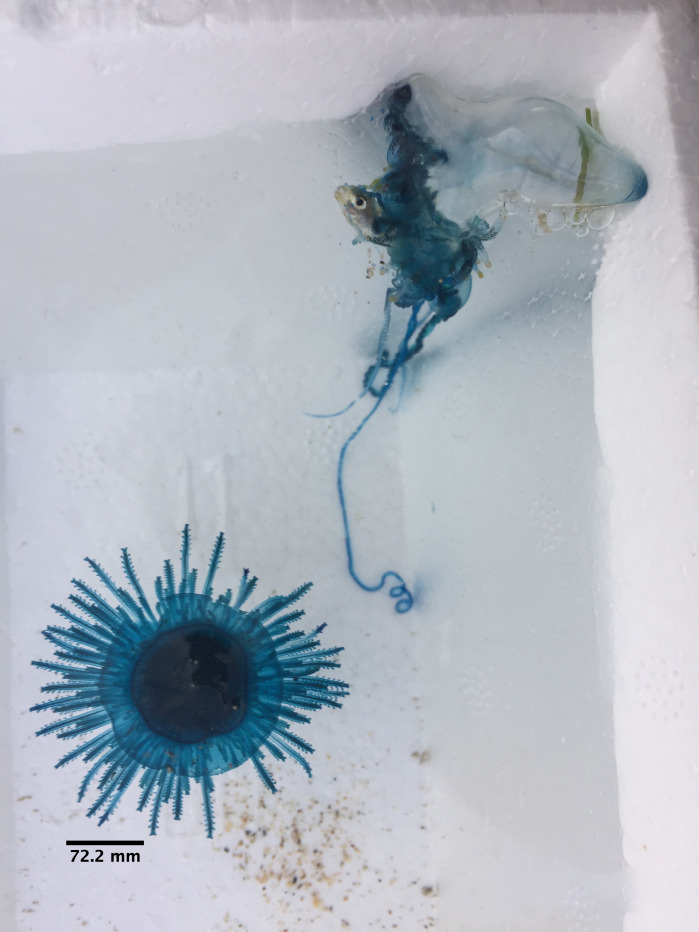
Stranded *Physaliaphysalis* (top) and *Porpitaporpita* (bottom) in Semawang Beach, Denpasar City, Bali Island.

**Figure 9.**
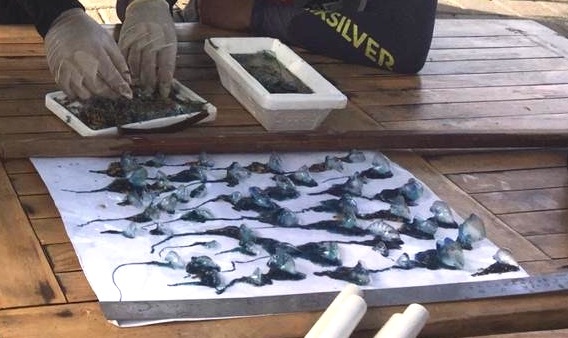
Counting and measuring individuals of the stranded *Physaliaphysalis* in the Semawang Beach, Denpasar City, Bali Island. The specimens' total lengths were 0.7-5.4 cm, measured from the top of the crest to the tip of the longest tentacle.

The specimen with medusa having a hemispherical shape has distinct black spots in the perradial bulbs and four radial canals could only be identified to the genus level as *Eucheilota*, based on Bouillon et al. (2006) (Fig. 2 and Fig. 3). The specimen with a hemispherical medusa, four radial canals, gonads located in the middle of the radial canals, 16 marginal tentacles with basal bulbs and cirri between the tentacles was identified as *Mitrocomella*, based on Schuchert (2017) and Bouillon et al. (2006) (Fig. 4 and Fig. 5). The species that is bright blue (adult) or yellow (young), disc-shaped, upper surface nearly flat, free-floating, no sail, a single mouth beneath the float, tentacles on the margin of the disc was identified as *Porpitaporpita* according to Schuchert (2010) (Fig. 6). Furthermore, *P.physalis* was identified following Totton (1960) as having a sail-shaped bluish-pinkish colour, free-floating and all zooids being submersible (Fig. 7). Both *Physaliaphysalis* and *Porpitaporpita* have a pneumatophore structure that allows them to float on the sea surface and comprise hydroid colonies (zooids) with specialised functions (Fig. 8). There were 114 individuals of *Physaliaphysalis* and ten individuals of *Porpitaporpita* collected, consisting of six blue and four yellow individuals. The collected *P.physalis* specimens measured 0.7-5.4 cm in length from the top of the crest to the tip of the longest tentacle (Fig. 9). Unlike the floating Hydromedusae, *Eucheilota* and *Mitrocomella* actively swim on the sea surface using the muscle contractions of the bell. The author did not count the exact number of *Eucheilota* and *Mitrocomella* as these organisms were spotted amongst ctenophores.

## Discussion

**Figure 10.**
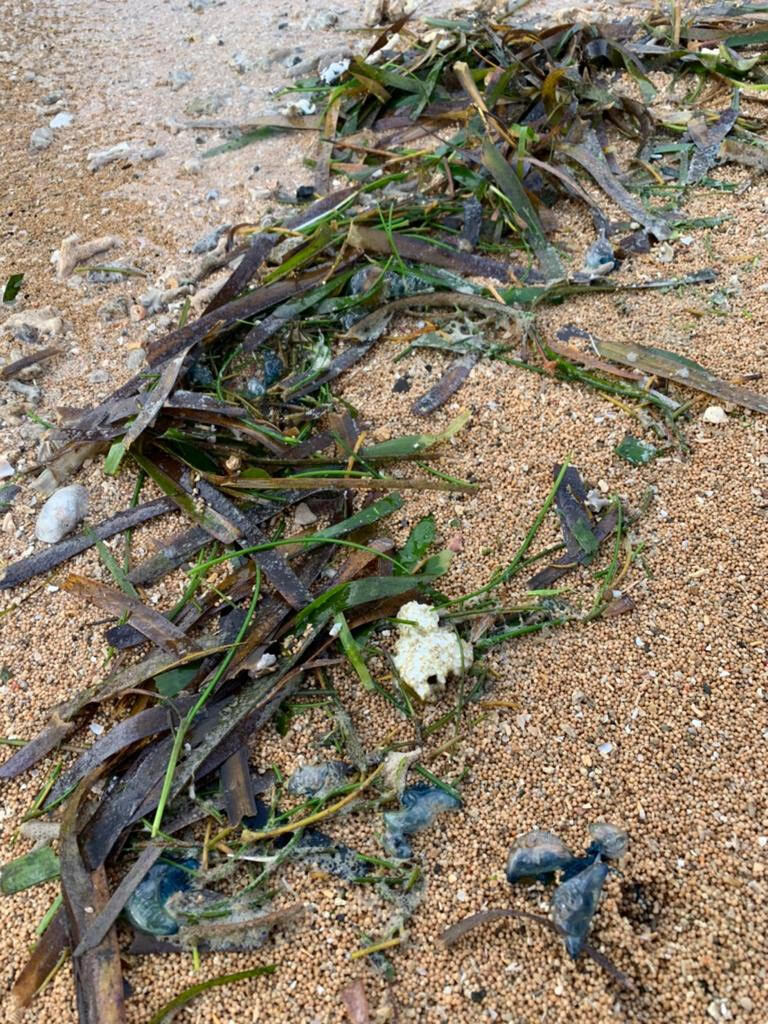
The massive stranding of *Physaliaphysalis* amongst the seagrass along the strand-line of Semawang Beach, Denpasar City, Bali Island.

According to the local people, *P.physalis* had been washed ashore since the end of June 2020, with each beach stranding event lasting approximately two weeks. Nevertheless, the observation conducted at the same site from June to August 2021 resulted in no stranded *Porpitaporpita* and *Physaliaphysalis* being seen. In addition, similar findings were also reported by the beach guards along the southern coasts of the Special Region of Yogyakarta Province, which has annual stinging cases of the stranded *P.physalis*, except in 2021. The period of strandings of *P.physalis* in these areas is the same as in the east Bali coasts. The local media in Bali started reporting the massive stranding of *P.physalis* (Fig. 10) since 2005 and it has become an annual phenomenon on the east coasts of Bali during June-August; however, there were no scientific data provided prior to this study. As pleuston, these organisms are driven by wind and ocean currents (Pandya et al. 2013). Thus their presence can be suggested to have been carried from somewhere along with the wind direction. There is south-easterly winds from the Australian continent to the equator during these months that reaches maximum wind speeds in June–August (Chang 2004, Chang et al. 2005, Wheeler and McBride 2005). This is also likely why these species have not been observed (nor reported by the fishermen and local people) in the northern part of Bali island.

*Mitrocomella* is abundant in plankton communities and can even become the major species in gelatinous plankton communities in the Arctic (Stepanjants 1989), temperate (Lock et al. 1999, Buecher and Gibbons 2000, Widmer 2004, Dutto et al. 2019) and tropical waters (Chunguang et al. 2018). As known in a few members of the genus, *Mitrocomella* can disperse in its life phases (medusae and hydroid), enabling it to occupy many marine environments. At the same time, the polyp of *Eucheilota* is highly tolerant to various environmental conditions, such as high salinity (Vannucci et al. 1970) and different temperatures (Altuna 2009). Thus, some of its members, including *E.menoni*, are invasive species in Atlantic and European waters (Altuna 2009). The observed medusa *Mitrocomella* in this study tend to be overlooked due to the white to transparent colour; in contrast, *Eucheilota* has noticeable medusae with four distinct black spots in the perradial bulbs. *Eucheilota* and *Mitrocomella* in Selini Beach in early April are presumably related to the relatively weak winds in these months due to the inter-monsoon periods in Indonesia (Chang 2004, Chang et al. 2005, Wheeler and McBride 2005), whereas other studies have revealed the appearances of these Hydromedusae during upwelling events (Pages et al. 1991; Miglietta et al. 2008). However, more investigation into the environmental effects that shape the dynamics of these Hydromedusae is needed. Despite the Hydromedusae in this report being native and common to tropical and subtropical seas in the Pacific, Atlantic and Indian seas (Bouillon and Barnett 1999, Zhang et al. 1999, Calder et al. 2003, Kirkendale and Calder 2003, Kubota and Tanase 2007,Calder 2010, Gul and Gravili 2014,Chunguang et al. 2018), data to indicate their presence in Bali waters, as well as in Indonesia waters, remain limited. Hence, to the best of the author's knowledge, this is the first scientific record of the observed occurrence of *Porpitaporpita*, *Physaliaphysalis*, *Eucheilota* and *Mitrocomella* in Bali waters.
